# A preliminary model for estimating mass-specific oxygen uptake during manual wheelchair propulsion from mechanical power and heart rate

**DOI:** 10.1371/journal.pone.0359323

**Published:** 2026-10-06

**Authors:** Mateusz Kukla, Michał Kończak, Maciej Berdychowski, Dominik Rybarczyk, Bartosz Wieczorek, Łukasz Warguła

**Affiliations:** 1 Institute of Machine Design, Faculty of Mechanical Engineering, Poznan University of Technology, Poznań, Poland; 2 Institute of Mechanical Technology, Faculty of Mechanical Engineering, Poznan University of Technology, Poznań, Poland; Polytechnic University of Guarda, PORTUGAL

## Abstract

Manual wheelchair users may have reduced physical activity and total energy expenditure, while wearable devices may not adequately capture external mechanical demand during wheelchair propulsion. This pilot study examined whether wheel-level mechanical power combined with heart rate could estimate mass-specific oxygen uptake (V̇O2/kg) during manual wheelchair propulsion. Ten long-term manual wheelchair users aged 29–38 years completed 3-min trials under three progressively loaded conditions on a wheelchair ergometer. Wheel velocity and braking torque were measured, mechanical power was calculated from trial-mean torque and angular velocity, and metabolic variables were assessed using a wearable respiratory gas analyzer. A two-predictor linear regression model was developed, with participant-clustered standard errors and leave-one-participant-out cross-validation (LOPO-CV) for internal evaluation. V̇O2/kg increased across conditions despite lower velocity under the highest load. The final model was V̇O_2_/kg = 5.5738 + 0.2444P + 0.03587HR. Mechanical power (95% CI: 0.1330–0.3558) and heart rate (95% CI: 0.01342–0.05833) were significant positive predictors. The model explained 72.2% of the variance ($R^{\text{2}}$ = 0.722; adjusted $R^{\text{2}}$ = 0.701). LOPO-CV yielded an RMSE of 1.128 mL O_2_·kg^−1^·min^−1^, an MAE of 0.917 mL O_2_·kg^−1^·min^−1^, and $R^{\text{2}}$ = 0.575. Repeated-measures Bland–Altman analysis of LOPO-CV predictions showed a mean bias of −0.156 mL O_2_·kg^−1^·min^−1^ (95% CI: −0.6259 to 0.2022), with limits of agreement from −2.389 (95% CI: −3.1667 to −1.7711) to 2.077 mL O_2_·kg^−1^·min^−1^ (95% CI: 1.6096 to 2.6636). Mechanical power and heart rate may provide complementary information for estimating metabolic demand during manual wheelchair propulsion. However, the model was developed in a small exploratory sample, was only internally evaluated, and requires external validation before practical application.

## 1. Introduction

People with disabilities constitute a population affected by substantial health disparities [1]. Consumer-level wrist-worn activity monitors have also shown limited accuracy at lower movement frequencies during wheelchair propulsion [2]. In individuals with SCI who use wheelchairs, reduced physical activity and the associated risk of obesity create a need for reliable information on activity-related energy expenditure [3]. Dedicated physical-activity monitoring systems have therefore been developed to estimate energy expenditure in manual wheelchair users [4]. Individuals with SCI may also exhibit reduced total energy expenditure because of altered body composition and lower levels of physical activity [5]. Sensor-based approaches to estimating energy expenditure in wheelchair users with SCI have consequently been investigated [6]. More broadly, disability-associated low-energy-expenditure deconditioning [7] and SCI-specific physical-activity guidelines [8] further emphasize the importance of appropriately monitoring and managing physical activity in this population.

It can be noted that the lack of dedicated reference values for individuals with SCI complicates the proper adjustment of energy and nutrient intake, thereby contributing to neurogenic obesity and cardiometabolic syndrome [5]. Findings from studies conducted in the general population indicate that behavioral weight loss interventions are effective and can lead to weight reduction among individuals with overweight or obesity [9]. Behavioral interventions aimed at modifying lifestyle are typically based on reducing energy intake, self-monitoring of body weight, diet, and physical activity (PA), as well as increasing energy expenditure (EE). Notably, recent studies suggest that combining behavioral interventions with technology designed to support self-monitoring yields superior outcomes. Such integrated approaches have been shown to result in greater weight loss compared to programs based solely on behavioral strategies [10].

A similar approach should be considered in the context of individuals using wheelchairs. The problem can be addressed through two complementary aspects: the development of methods and devices for monitoring physical activity, and the advancement of methods and techniques for estimating energy requirements in individuals who regularly use manual wheelchairs.

It is important to highlight the consequences of underestimating or overestimating the energy requirements of individuals in this population, which primarily result from reduced muscle mass and neuroendocrine alterations [11,12]. For instance, study [13] reported that total daily energy expenditure (TDEE) in persons with SCI is approximately 1870 kcal/day, compared to 2376 kcal/day in a nondisabled control group. This discrepancy may contribute to the development of neurogenic obesity and metabolic complications. Predictive equations commonly used in the general population tend to overestimate the energy requirements of individuals with SCI. This observation supports the need for SCI-specific formulations that account for the level of injury and training regimen. Consequently, individualized approaches to estimating energy expenditure are required, preferably based on indirect calorimetry [5].

The development of digital technologies such as smartwatches and smartphones enables users to monitor physical activity and estimate energy expenditure. In manual wheelchair users, energy-expenditure prediction has been investigated using the RT3 accelerometer [14], the SenseWear Armband [15], and more recent wearable systems combining physiological and inertial-sensor data [16]. The physiological determinants of energy expenditure differ in SCI populations [17], whereas many accelerometer-based prediction approaches have been developed in ambulatory adults without disability [18]. Commercial wrist- and arm-worn devices also show activity- and device-dependent errors in energy-expenditure estimation [19,20]. These limitations motivate the development of wheelchair-specific monitoring approaches. Wheelchair-mounted sensing has been investigated as one such approach [21], and a comprehensive review of technologies for measuring manual-wheelchair propulsion metrics is available in [22].

Most existing approaches estimate energy expenditure from kinematic signals recorded by upper-limb-worn sensors or from heart rate alone; however, these variables may not fully capture changes in the external mechanical demand of wheelchair propulsion. Mechanical power, calculated from propulsion torque and wheel velocity, provides a direct measure of external work rate and may therefore distinguish between conditions involving similar propulsion velocities but different mechanical loads. Heart rate provides complementary information on the user’s physiological response to this external workload. Accordingly, combining mechanical power and heart rate may provide a means of capturing both external mechanical demand and physiological response; however, this combination remains insufficiently investigated during manual wheelchair propulsion under controlled, incrementally loaded conditions.

Accordingly, the primary aim of this pilot study was to identify the general form and direction of the relationship between metabolic demand and selected propulsion-related variables by developing a preliminary mathematical model for estimating mass-specific oxygen uptake (V̇O_2_/kg, mL O_2_·kg^−1^·min^−1^) during manual wheelchair propulsion based on mechanical power and heart rate. More specifically, the study examined whether wheel-level mechanical power provides information complementary to heart rate when estimating the metabolic demand of manual wheelchair propulsion under different load conditions. Because MET values are defined relative to resting oxygen uptake, the estimated V̇O_2_/kg values can subsequently be expressed as standard MET by dividing them by 3.5 mL O_2_·kg^−1^·min^−1^ or, where appropriate for comparisons with SCI-specific populations, as SCI MET by dividing them by 2.7 mL O_2_·kg^−1^·min^−1^. Thus, mass-specific oxygen uptake provides a directly measured metabolic outcome without requiring the application of a population-specific resting metabolic equivalent, while retaining comparability with MET-based values commonly reported in the literature.

## 2. Methods

### 2.1 Participants

A total of 10 volunteers participated in the study, including both males and females aged between 29 and 38 years. Inclusion criteria comprised all reasons for wheelchair use, provided that participants had used a wheelchair for at least 20 hours per week for a minimum of 10 years. Exclusion criteria included the inability to use a manual wheelchair, specifically in individuals with a spinal cord injury at the level of C8 or above, as well as the presence of upper limb joint or muscle disorders. Participants were also excluded if they had any illness or injury that could interfere with their participation in the planned experimental procedures, particularly respiratory infections that could affect metabolic measurements. The study received ethical approval from the Bioethics Committee at the K. Marcinkowski Medical University in Poznań (resolution no. 513/21 of 24 June 2021). The recruitment period for this study extended from early March to the end of October 2024. The study group consisted of 7 males and 3 females, with only one individual presenting incomplete paralysis. All participants were informed of the study objectives and experimental procedures, and written informed consent was obtained prior to participation. Furthermore, participants were required to submit a written declaration of their health status and official documentation confirming their disability status. Information regarding the level and completeness of the lesion was self-reported. Demographic characteristics of the participants are presented in Table 1. A table providing detailed characteristics of the individual study participants has been included in S1 Table. All participants received financial compensation for their participation in accordance with the regulations governing the funded research project. Because the experiment described in this manuscript formed part of a broader project involving several wheelchair-propulsion studies, compensation was calculated individually according to the scope of the completed procedures, the time devoted to participation, and the number of laboratory visits. The compensation was unrelated to participant performance, physiological measurements, or study outcomes. Participation was voluntary, and participants retained the right to decline individual procedures or withdraw from the study at any time without penalty; compensation was based only on the time and procedures completed. No a priori sample-size calculation was performed because the study was designed as an exploratory pilot investigation; accordingly, the present sample supports preliminary model development and internal evaluation only.

**Table 1:** General characteristics of participants.

	Mean ± SD
Sex (M/F)	7/3
Age (years)	33.6 ± 3.7
Duration of disability (years)	26.9 ± 7.9
Height (cm)	171.3 ± 10.8
Weight (kg)	83.7 ± 20.8

### 2.2 Experimental equipment

The study utilized a measurement setup based on a custom-designed wheelchair ergometer equipped with sensors enabling the measurement of angular velocity using an HY38–500 incremental encoder (Termipol, Lubliniec, Poland). The encoder provided 500 pulses per revolution. Angular velocity was calculated from the number of pulses recorded within each sampling interval and was independently verified using a PCE-T 230 contact tachometer (PCE Deutschland GmbH, Meschede, Germany), with a measurement error below 0.8%. Braking torque was measured using a T22/100NM torque transducer (Hottinger Brüel & Kjaer GmbH, Darmstadt, Germany). The torque transducer was factory calibrated, with a manufacturer-specified accuracy class of 0.5. Data acquisition was performed using a dedicated software application developed in the Python programming language. A detailed description of the wheelchair dynamometer design can be found in [23]. Metabolic parameters were assessed using a wearable breathing gas analyzer (K5, COSMED, Italy), which was integrated with a heart rate monitoring device (smartLAB hrm W, HMM Diagnostics GmbH, Heddesheim, Germany). Data acquisition from these devices was conducted using dedicated OMNIA 2.1 software (COSMED, Rome, Italy). Before each set of measurements, the K5 system underwent ambient-air calibration, reference-gas calibration using a certified gas mixture containing 16% O_2_ and 5% CO_2_, with N_2_ as the balance gas, and turbine calibration using a COSMED 3-L Calibration Syringe (PN C00600-01-11). Calibration was performed after the device had completed its warm-up procedure, as the system incorporates an electrochemical sensor whose response characteristics depend on operating temperature*.* Gas exchange variables were recorded on a breath-by-breath basis. For the present analysis, metabolic variables were averaged over the complete 3-min experimental trial. No prospectively defined steady-state criterion was applied. This approach is recommended by the COSMED protocol and has also been applied in previous studies utilizing this device [24,25].

During the experiments, participants used their personal, everyday wheelchairs. Although the wheelchairs differed in model and manufacturer, all were active manual wheelchairs with a mass of 12 kg or less and 24-inch wheels. Tire pressure was set to the nominal pressure specified by the manufacturer for each individual tire. Wheelchair configurations were otherwise not standardized, as participants used their habitual wheelchair setups. This approach was adopted because, as indicated in [26,27], differences in such parameters, including weight, are reflected in oxygen uptake values.

### 2.3 Experimental procedure

Prior to testing, calibration of the air, gas, and turbine of the wearable respiratory gas analyzer was performed in accordance with the manufacturer’s guidelines. Subsequently, the participant was seated in a wheelchair secured on the wheelchair dynamometer. The participant was then allowed a familiarization period of up to 5 minutes, during which they could perform trial propulsion without additional load in order to adapt to the setup. Following this period, the participant was fitted with the respiratory gas analyzer. The experimental protocol consisted of 3-minute wheelchair propulsion trials, during which participants were instructed to generate push strokes in synchrony with a metronome set at 30 BPM. As a result, the propulsion cycle frequency was standardized across participants, while the resulting average velocity was treated as an outcome parameter. Consequently, mean velocity varied between participants and was intentionally allowed to remain self-selected in order to avoid excessive loading and artificial modification of individual propulsion technique, while enabling participants to adapt their propulsion speed naturally to the imposed resistance according to their physical capacity and fitness level. Between experimental conditions, participants remained seated in their wheelchairs while respiratory quotient (RQ) and heart rate (HR) were monitored. The subsequent trial was initiated when RQ decreased below 0.8 and HR had sufficiently decreased from the level observed during the preceding exercise bout. If these conditions were not met, the participant continued to rest and the parameters were reassessed after an additional period. No predefined numerical HR recovery criterion was used, and individual recovery durations were not prospectively recorded.

Measurements were conducted under three conditions, denoted as $M_{\text{1}},M_{\text{2}},M_{\text{3}}$, corresponding to different levels of load torque. The varying parameter was the braking torque $M_{B}$, defined such that $M_{\text{1}}<M_{\text{2}}<M_{\text{3}}$. The lowest value, $M_{\text{1}}$, resulted from inherent system friction and internal resistance. Higher torque levels were achieved by introducing additional braking torque using a disc brake, in accordance with relationships (1) and (2):


$$\begin{array}{c} M_{\text{2}}=M_{\text{1}}+M_{B\text{1}} \end{array}$$ (1)

$$\begin{array}{c} M_{\text{3}}=M_{\text{1}}+M_{B\text{2}} \end{array}$$ (2)

The values of braking torque $M_{B\text{1}}$ and $M_{B\text{2}}$ were kept constant for all participants and across all experimental conditions. However, the resulting load torque values $M_{\text{1}},M_{\text{2}},M_{\text{3}}$ differed between participants. This was due to their dependence on system resistances, not all of which remained constant (e.g., frictional forces vary, among other factors, with the participant’s body weight). As a consequence, exercise intensity increased gradually in three steps, while the load experienced by each participant differed. During the experiments, the following parameters were measured: oxygen uptake V̇O_2_ (mL/min), carbon dioxide production V̇CO_2_ (mL/min), mass-specific oxygen uptake (V̇O_2_/kg, mL O_2_·kg^−1^·min^−1^), energy expenditure per minute EEm (kcal/min), heart rate HR (BPM), wheelchair wheel angular velocity n (RPM), and load torque $M_{\text{1}-\text{3}}$ (Nm).

### 2.4 Data processing and analysis

Data recorded using the measurement devices were transferred to an Excel spreadsheet (Microsoft Excel, Microsoft Corp., Redmond, WA, USA). The datasets of angular velocity n (RPM) and load torque M (Nm) were subjected to low-pass filtering using a fourth-order Butterworth filter with a cutoff frequency of 10 Hz [28,29]. Based on the angular velocity of the wheelchair wheel, the corresponding linear velocity v (km/h) was determined. Trial-mean values were subsequently calculated for each participant and experimental condition. Descriptive data are presented as mean ± standard deviation (SD). All statistical analyses were performed using MATLAB (MathWorks, Natick, MA, USA), with the level of statistical significance set at p < 0.05.

Differences between the three experimental conditions ($M_{\text{1}}$, $M_{\text{2}}$ and $M_{\text{3}}$) were assessed using one-way repeated-measures analysis of variance (ANOVA), with experimental condition treated as a within-subject factor. Greenhouse–Geisser-corrected results were reported to account for potential violations of sphericity. For V̇O_2_/kg and mean velocity, significant omnibus effects were followed by paired-samples comparisons with Holm correction for multiple testing. Partial eta squared ($\eta_{p}^{\text{2}}$) was reported as the effect size for repeated-measures ANOVA, whereas Cohen’s $d_{z}$ and 95% confidence intervals for paired mean differences were reported for pairwise comparisons.

Mass-specific oxygen uptake (V̇O_2_/kg, mL O_2_·kg^−1^·min^−1^), measured using indirect calorimetry, was used as the primary metabolic outcome. The SCI-specific MET conversion was not applied to the entire study group because the participants represented different underlying causes of mobility impairment and spinal cord injury was not confirmed in all cases. SCI MET is a linear transformation of mass-specific oxygen uptake, defined as SCI MET = (V̇O_2_/kg)/2.7, while V̇O_2_/kg can be obtained as SCI MET × 2.7. Therefore, replacing SCI MET with directly measured V̇O_2_/kg does not alter the underlying relationships between the variables but avoids applying an SCI-specific normalization factor to a heterogeneous study population. For comparisons with previous studies, metabolic outcomes were reported in the form provided by the original publications, because the definitions of MET, physical activity energy expenditure, and total energy expenditure were not consistent across studies and therefore could not be reliably converted to a common V̇O_2_/kg scale.

Simple linear regression analyses were performed separately for the relationships between V̇O_2_/kg and mean wheelchair velocity and between mean propulsion torque and mean wheelchair velocity. Subsequently, a two-predictor multiple linear regression model was developed to estimate V̇O_2_/kg from mechanical power and heart rate. Mechanical power was calculated from trial-mean propulsion torque and trial-mean wheel angular velocity. Because each participant contributed three observations, statistical inference for the model coefficients was based on participant-clustered standard errors with a small-sample correction to account for within-participant dependence. Model diagnostics included inspection of residual-versus-fitted and quantile–quantile plots, assessment of heteroscedasticity using the Breusch–Pagan test, and assessment of multicollinearity using variance inflation factors (VIF). No observations were excluded on the basis of regression diagnostics.

To assess the sensitivity of the model to inclusion of the initial oxygen-uptake transient, an additional analysis was performed using variables averaged over the final 30 s of each experimental trial. Mechanical power was calculated from the resulting mean torque and mean wheel angular velocity. The same multiple-regression, participant-clustered inference, LOPO-CV, and repeated-measures Bland–Altman procedures as in the primary analysis were then repeated using these final-30-s values.

Internal predictive performance was evaluated using participant-level leave-one-participant-out cross-validation (LOPO-CV). In each of the ten folds, all three observations from one participant were excluded from model development, the model was fitted using data from the remaining nine participants, and predictions were generated for the excluded participant. Predictive performance was quantified using the root mean squared error (RMSE), mean absolute error (MAE), and cross-validated coefficient of determination ($R^{\text{2}}$). Agreement between measured and LOPO-CV-predicted V̇O_2_/kg values was additionally assessed using repeated-measures Bland–Altman analysis accounting for repeated observations within participants. Ninety-five percent confidence intervals for the mean bias and limits of agreement were estimated using participant-level bootstrap resampling with 10,000 iterations.

## 3. Results

The experiments were conducted under three conditions, each corresponding to increasing levels of load torque ($M_{\text{1}}<M_{\text{2}}<M_{\text{3}}$). In the first condition, the mean velocity was 1.8 ± 0.8 km/h, with a mass-specific oxygen uptake of 9.0 ± 1.7 mL O_2_·kg^−1^·min^−1^, while energy expenditure per minute was 3.4 ± 0.6 kcal/min. In the second condition, the mean velocity was 1.8 ± 0.8 km/h, with a V̇O_2_/kg value of 10.2 ± 1.5 mL O_2_·kg^−1^·min^−1^, and energy expenditure per minute was 3.9 ± 0.5 kcal/min. Finally, in the third condition, the mean velocity was 1.5 ± 0.8 km/h, while V̇O_2_/kg was 11.3 ± 1.5 mL O_2_·kg^−1^·min^−1^, and energy expenditure per minute was 4.3 ± 0.6 kcal/min. For the analyzed conditions, the mean load torque values were 1.9 ± 0.4 Nm, 2.3 ± 0.7 Nm, and 3.4 ± 1.5 Nm, respectively. A summary of the obtained results is presented in Table 2. The table also includes values of relative change for individual parameters in the second and third conditions relative to the first condition, i.e., ${\Delta (M}_{\text{2}}-M_{\text{1}})$ and $\Delta (M_{\text{3}}-M_{\text{1}})$, respectively. Relative changes for those conditions were calculated from the corresponding group mean values using condition $M_{\text{1}}$ as the reference: Δ = ($M_{x}$−$M_{\text{1}}$)/$M_{\text{1}}$ ×100%, where $M_{x}$ denotes condition $M_{\text{2}}$ or $M_{\text{3}}$. For each experimental condition, mechanical power P was calculated from trial-mean torque and trial-mean angular velocity, and relative mechanical power $P_{rel}$ was subsequently obtained by dividing mechanical power by the participant’s body mass. A table providing detailed characteristics of the individual participant-level mechanical parameters has been included in S2 Table.

**Table 2:** A summary of the obtained results.

Variant	$M_{1}$ mean ± SD	$M_{2}$ mean ± SD	$M_{3}\;$mean ± SD	$M_{2}-M_{1}$ (%)	$M_{3}-M_{1}$ (%)
M (Nm)	1.88 ± 0.44	2.26 ± 0.65	3.36 ± 1.48	20.2	78.6
v (km/h)	1.77 ± 0.77	1.78 ± 0.80	1.52 ± 0.77	0.6	−13.8
P (W)	3.27 ± 2.10	4.07 ± 2.98	5.43 ± 5.21	24.5	66.3
$P_{rel}$ (W/kg)	0.0369 ± 0.0167	0.0453 ± 0.0235	0.0582 ± 0.0407	22.8	57.7
V̇O_2_/kg (mL O_2_·kg^−1^·min^−1^)	9.00 ± 1.68	10.19 ± 1.49	11.27 ± 1.45	13.2	25.2
HR (BPM)	96.39 ± 22.88	99.05 ± 21.37	100.81 ± 19.25	2.8	4.6
EEm (kcal/min)	3.43 ± 0.58	3.88 ± 0.51	4.30 ± 0.56	13.2	25.4

Repeated-measures analysis demonstrated a significant effect of experimental condition on load torque (F(1.20, 10.81) = 14.75, p = 0.002, $\eta_{p}^{\text{2}}$ = 0.621), mean velocity (F(1.33, 11.93) = 9.07, p = 0.007, $\eta_{p}^{\text{2}}$ = 0.502), V̇O_2_/kg (F(1.98, 17.86) = 71.50, p < 0.001, $\eta_{p}^{\text{2}}$ = 0.888), EEm (F(1.88, 16.90) = 64.08, p < 0.001, $\eta_{p}^{\text{2}}$ = 0.877). No significant effect of condition was observed for HR (F (1.97, 17.69) = 0.36, p = 0.699, $\eta_{p}^{\text{2}}$ = 0.038). Pairwise comparisons demonstrated that V̇O_2_/kg increased significantly between all consecutive conditions: by 1.187 mL O_2_·kg^−1^·min^−1^ between $M_{\text{1}}$ and $M_{\text{2}}$ (95% CI [0.774, 1.600], $d_{z}$ = 2.06, p < 0.001), and by a further 1.080 mL O_2_·kg^−1^·min^−1^ between $M_{\text{2}}$ and $M_{\text{3}}$ (95% CI [0.634, 1.526], $d_{z}$ = 1.73, p < 0.001). Pairwise comparisons showed no significant difference in mean velocity between $M_{\text{1}}$ and $M_{\text{2}}$ (mean difference = 0.010 km/h, 95% CI: −0.110 to 0.130, Cohen’s $d_{z}$ = 0.06, Holm-adjusted p = 0.852). Mean velocity was significantly lower in $M_{\text{3}}$ than in $M_{\text{1}}$ (mean difference = −0.244 km/h, 95% CI: −0.370 to −0.119, Cohen’s $d_{z}$ = −1.39, Holm-adjusted p = 0.0052) and $M_{\text{2}}$ (mean difference = −0.254 km/h, 95% CI: −0.454 to −0.054, Cohen’s $d_{z}$ = −0.91, Holm-adjusted p = 0.0367).

The plot presented in Fig 1 illustrates the relationship between mass-specific oxygen uptake and mean wheelchair propulsion velocity for each experimental condition. Significant positive correlations between V̇O_2_/kg and mean velocity were observed in all three conditions ($M_{\text{1}}$: r = 0.905, p = 0.0003; $M_{\text{2}}$: r = 0.899, p = 0.0004; $M_{\text{3}}$: r = 0.931, p < 0.0001). Condition-specific linear regression analysis yielded the following relationships: $M_{\text{1}}$, V̇O_2_/kg = 5.5273 + 1.9673v (intercept 95% CI: 4.0865 to 6.9680; slope 95% CI: 1.2140 to 2.7205; $R^{\text{2}}$ = 0.819); $M_{\text{2}}$, V̇O_2_/kg = 7.2354 + 1.6628v (intercept 95% CI: 5.9591 to 8.5116; slope 95% CI: 1.0029 to 2.3228; $R^{\text{2}}$ = 0.808); and $M_{\text{3}}$, V̇O_2_/kg = 8.5963 + 1.7560v (intercept 95% CI: 7.6464 to 9.5463; slope 95% CI: 1.1935 to 2.3185; $R^{\text{2}}$ = 0.866).The plot presented in Fig 2 illustrates the relationship between mean propulsion torque and mean wheelchair velocity for all experimental conditions. Significant positive correlations were observed in each condition ($M_{\text{1}}$: r = 0.834, p = 0.0027; $M_{\text{2}}$: r = 0.935, p < 0.0001; $M_{\text{3}}$: r = 0.818, p = 0.0038). The corresponding linear regression equations were: $M_{\text{1}}$, M = 1.0323 + 0.4806v (intercept 95% CI: 0.5366 to 1.5281; slope 95% CI: 0.2214 to 0.7398; $R^{\text{2}}$ = 0.696); $M_{\text{2}}$, M = 0.9152 + 0.7575v (intercept 95% CI: 0.4623 to 1.3681; slope 95% CI: 0.5233 to 0.9917; $R^{\text{2}}$ = 0.874); and $M_{\text{3}}$, M = 0.9568 + 1.5778v (intercept 95% CI: −0.5709 to 2.4845; slope 95% CI: 0.6731 to 2.4825; $R^{\text{2}}$ = 0.669). For descriptive comparison, Table 3 presents propulsion velocities and energy-expenditure-related outcomes reported in previous wheelchair-propulsion studies alongside the values obtained in the present study. Because the definitions of the reported metabolic outcomes differed between studies, these values should not be interpreted as directly equivalent physiological measures.

**Table 3:** Descriptive comparison of propulsion velocity and metabolic outcomes reported in previous wheelchair-propulsion studies.

Source	v (km/h)	Reported energy-related value (kcal/min)	SD (kcal/min)	Metabolic measure
[30]	3	1.9	0.4	PAEE*
	4	2.4	0.6	PAEE*
	5	3.2	1	PAEE*
	6	4.3	1.7	PAEE*
	7	4.7	0.9	PAEE*
[31]	3	1.9	0.4	PAEE*
	4	2.4	0.6	PAEE*
	5	3.1	1	PAEE*
	6	4.2	1.7	PAEE*
	7	4.7	0.9	PAEE*
[32]	2	1.3**	0.4**	Metabolic rate
	4	2.4**	0.9**	Metabolic rate
	6	3.8**	1.0**	Metabolic rate
	8	5.9**	1.9**	Metabolic rate
[33]	3***	2.02	0.2	EE
	4***	2.6	0.4	EE
	5***	2.9	0.4	EE
[3]	2.2	3.2	0.8	EE
	3.5	4.3	0.9	EE
	5.1	6	1.2	EE
Present study^+^	1.8 ($M_{\text{1}}$)	3.4	0.6	EEm
	1.8 ($M_{\text{2}}$)	3.9	0.5	EEm
	1.5 ($M_{\text{3}}$)	4.3	0.6	EEm

* PAEE denotes physical activity energy expenditure rather than total energy expenditure. In [31], reported MET values were calculated by dividing oxygen uptake during each activity by individually measured resting oxygen uptake and were therefore not converted to V̇O_2_/kg using a fixed resting metabolic equivalent.

** Values were converted from kJ/min to kcal/min using the relationship EE (kcal/min) = 0.239 × EE (kJ/min).

*** The table includes the upper bounds of the velocity ranges.

+ ${𝐌}_{1}-{𝐌}_{3}$ denote three experimental conditions with progressively increasing load torque. Direct numerical comparisons with previous studies should be interpreted cautiously because of differences in participant characteristics, wheelchair configuration, propulsion protocols, and methods of prescribing exercise intensity and definitions of the reported metabolic outcomes.

**Fig 1:**
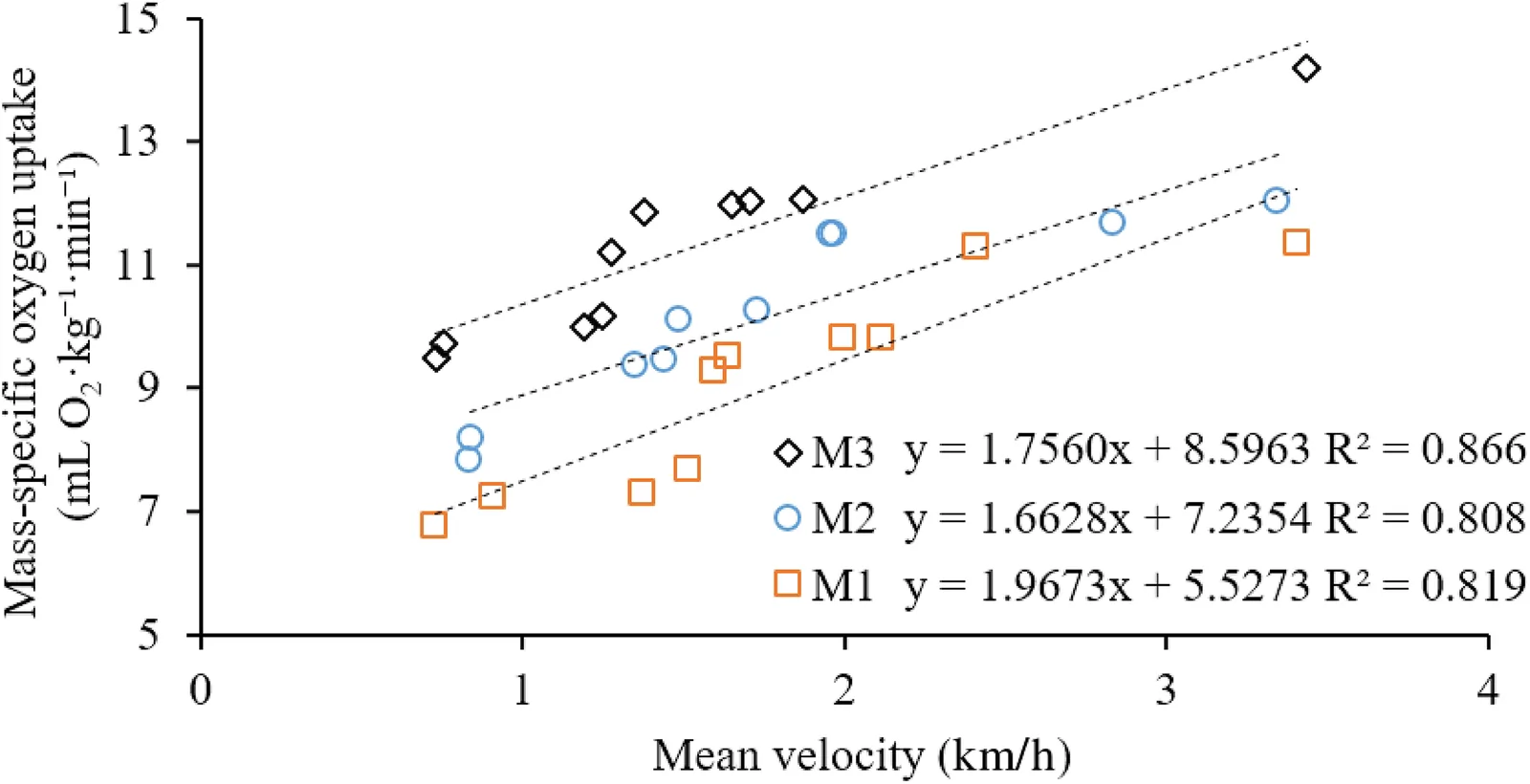
Change in V̇O_2_/kg value as a function of mean velocity for test variants $M_{1},M_{2},M_{3}$.

**Fig 2:**
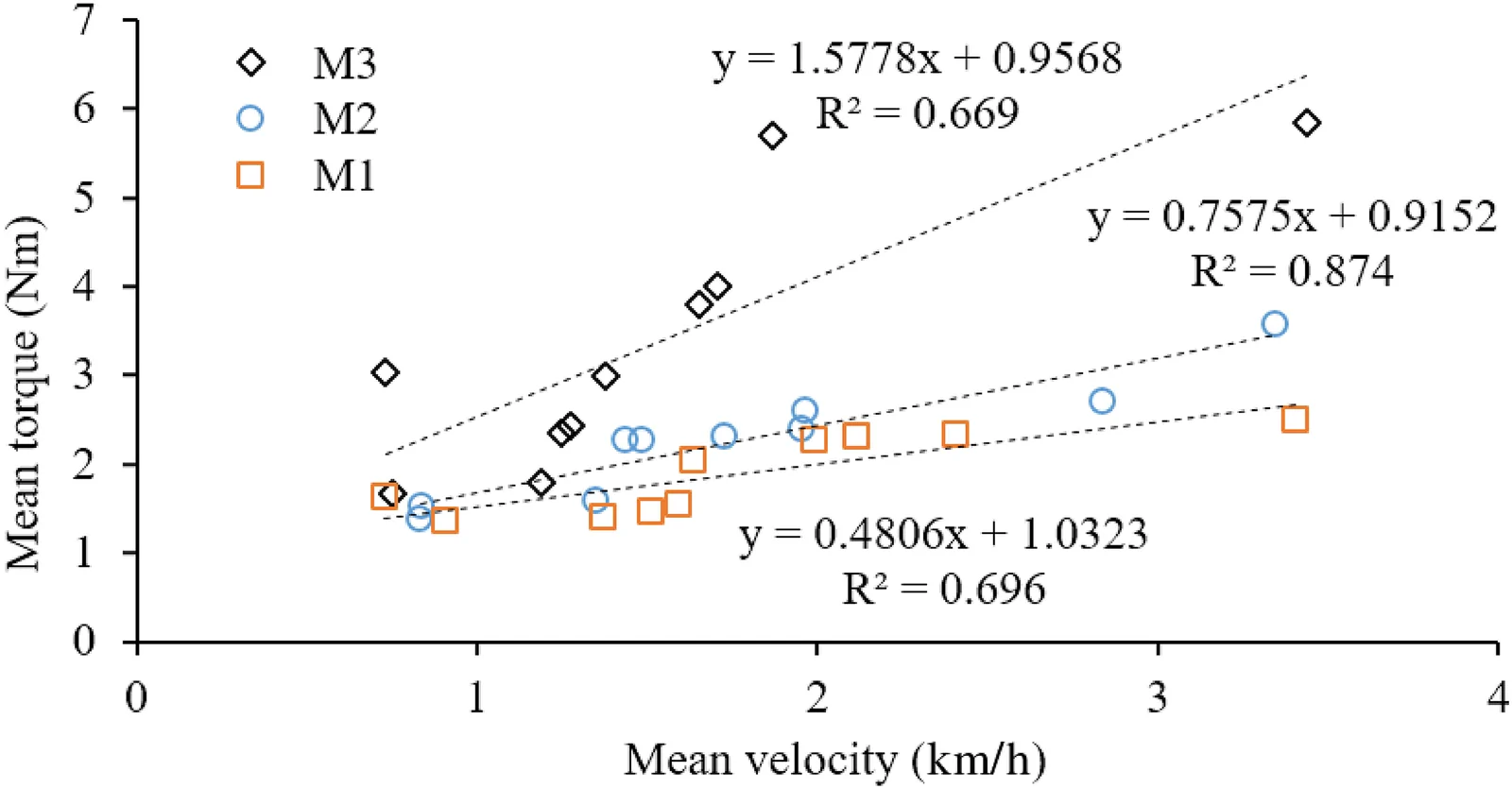
Change in mean torque value as a function of mean velocity for test variants $M_{1},M_{2},M_{3}$.

The observed relationships between torque and velocity were condition- and participant-dependent and should not be interpreted as a general physiological relationship. Mechanical power was used in the subsequent analysis primarily on biomechanical grounds, as the product of propulsion torque and angular velocity represents the external rate of mechanical work performed during wheelchair propulsion and incorporates both propulsion speed and the mechanical resistance that must be overcome. An additional advantage of this data representation is the possibility of comparing the obtained results with those reported in other experimental studies.

Heart rate was included as a physiological predictor complementary to mechanical power. Therefore, a multiple linear regression analysis was performed using two predictor variables: mechanical power P (W) and heart rate (HR, BPM), as described by equation (4):


$$\begin{array}{c} {\dot{V}O}_{\text{2}}/kg\;(mL\;O_{\text{2}}\cdot kg^{-\text{1}}\cdot min^{-\text{1}})=\;a\;+\;b\times \;(mechanical\;power,\;W)\;+\;c\times (heart\;rate,\;BPM). \end{array}$$ (4)

In the above equation, the values a, b, and c represent the model coefficients and were determined as follows: a = 5.5738, b = 0.2444, and c = 0.03587. Using participant-clustered standard errors, the intercept was 5.5738 (SE = 0.9234, 95% CI: 3.4850 to 7.6626, p = 0.0002), the coefficient for mechanical power was 0.2444 (SE = 0.0492, 95% CI: 0.1330 to 0.3558, p = 0.0008), and the coefficient for heart rate was 0.03587 (SE = 0.00993, 95% CI: 0.01342 to 0.05833, p = 0.0056). Thus, both mechanical power and heart rate were significant positive predictors of V̇O_2_/kg. The overall cluster-robust model was statistically significant, F(2,9) = 59.18, p < 0.0001, and explained 72.2% of the variance in the development dataset ($R^{\text{2}}$ = 0.722; adjusted $R^{\text{2}}$ = 0.701), with an apparent RMSE of 0.912 mL O_2_·kg^−1^·min^−1^ and an MAE of 0.747 mL O_2_·kg^−1^·min^−1^. To account for the repeated-measures design and to evaluate internal predictive performance, participant-level leave-one-participant-out cross-validation (LOPO-CV) was additionally performed. This yielded an RMSE of 1.128 mL O_2_·kg^−1^·min^−1^, an MAE of 0.917 mL O_2_·kg^−1^·min^−1^, and a cross-validated $R^{\text{2}}$ of 0.575, indicating moderate out-of-sample predictive performance. Fig 3 presents the repeated-measures Bland–Altman analysis of measured and participant-level LOPO-CV-predicted V̇O_2_/kg values, which was used to assess systematic bias and the limits of agreement while accounting for repeated observations within participants. Regression diagnostics did not indicate substantial heteroscedasticity (Breusch–Pagan $\chi^{\text{2}}$(2) = 2.91, p = 0.233) or problematic multicollinearity (VIF = 1.85 for both predictors). Visual inspection of the residual-versus-fitted and Q–Q plots did not reveal marked deviations from the regression assumptions.

**Fig 3:**
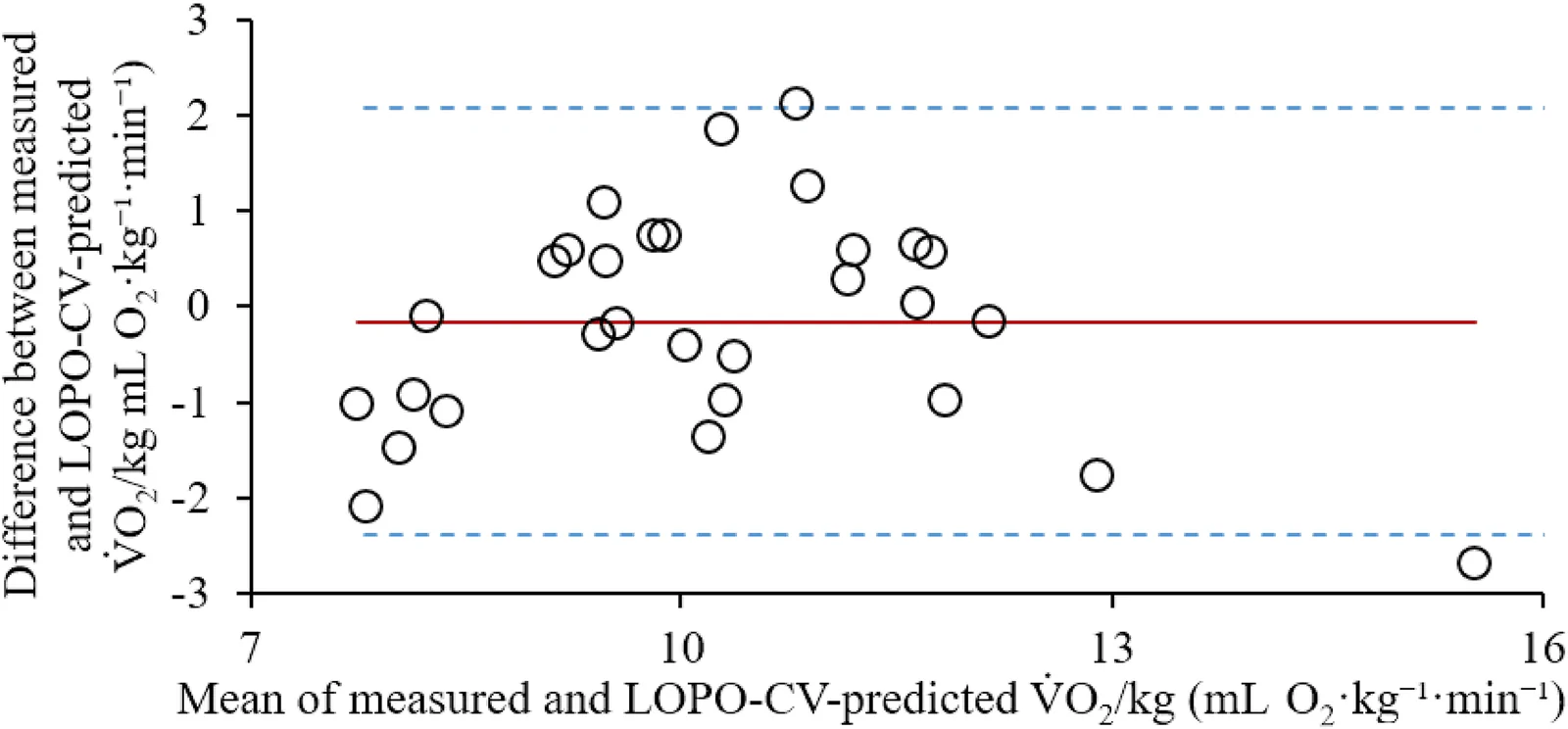
Repeated-measures Bland–Altman plot comparing measured V̇O_2_/kg values with participant-level leave-one-participant-out cross-validation (LOPO-CV) predictions. The solid line represents the mean bias, and the dashed lines represent the 95% limits of agreement.

As a sensitivity analysis, the model was recalculated using variables averaged over the final 30 s of each trial. Mean V̇O_2_/kg values for the final 30 s were 9.93 ± 2.10, 11.64 ± 2.02, and 12.95 ± 1.69 mL O_2_·kg^−1^·min^−1^ for $M_{\text{1}}$, $M_{\text{2}}$, and $M_{\text{3}}$, respectively. The resulting model was V̇O_2_/kg = 4.9025 + 0.1789P + 0.05743HR. Mechanical power (95% CI: 0.0444 to 0.3134, p = 0.0148) and heart rate (95% CI: 0.02897 to 0.08590, p = 0.0014) remained significant positive predictors. The model explained 62.8% of the variance in the development dataset ($R^{\text{2}}$ = 0.628; adjusted $R^{\text{2}}$ = 0.601). LOPO-CV yielded an RMSE of 1.444 mL O_2_·kg^−1^·min^−1^, an MAE of 1.244 mL O_2_·kg^−1^·min^−1^, and a cross-validated $R^{\text{2}}$ of 0.578. Repeated-measures Bland–Altman analysis yielded a mean bias of 0.048 mL O_2_·kg^−1^·min^−1^ and limits of agreement from −3.090 to 3.185 mL O_2_·kg^−1^·min^−1^. Thus, although use of the final 30 s resulted in higher mean V̇O_2_/kg values and altered the model coefficients, the principal finding that mechanical power and heart rate provide complementary predictive information was retained, and cross-validated $R^{\text{2}}$ was nearly unchanged compared with the primary whole-trial analysis (0.578 vs 0.575).

## 4. Discussion

A previous study reported a linear relationship between wheelchair propulsion velocity and MET-based estimates of metabolic demand [3]. Previous studies [30–32] have also demonstrated systematic changes in energy-expenditure-related outcomes with increasing wheelchair propulsion velocity, although the metabolic variables and their definitions differed between studies. Therefore, regression analysis was performed in the present study to assess the relationship between mean wheelchair velocity and directly measured V̇O_2_/kg. Analysis of the presented data, together with the coefficient of determination ($R^{\text{2}}$), indicates the existence of a strong linear relationship between the analyzed variables. Depending on the experimental condition ($M_{\text{1}},M_{\text{2}}$ and $M_{\text{3}}$), the mean velocities were 1.8 ± 0.8 km/h, 1.8 ± 0.8 km/h, and 1.5 ± 0.8 km/h, respectively. Consequently, the percentage differences between the second ($M_{\text{1}}/M_{\text{2}}$) and third ($M_{\text{1}}/M_{\text{3}}$) conditions relative to the first condition were 0.6% and −13.8% (decrease in mean velocity), respectively. As can be observed, the mean velocity did not differ substantially between the first and second conditions. However, a noticeable reduction in mean velocity was observed in the third condition. This was most likely caused by increased muscular load, as the highest load torque value was applied in this condition. This observation appears noteworthy and requires further investigation.

Analysis of Table 2 indicates that the recorded propulsion velocities were within the lower range of those reported in previous studies, where mean velocities of approximately 2 km/h or higher were commonly investigated. Mean velocity was similar in conditions $M_{\text{1}}$ and $M_{\text{2}}$, whereas a noticeable decrease was observed in $M_{\text{3}}$. Importantly, however, V̇O_2_/kg and energy expenditure increased progressively across the three conditions despite the reduction in velocity under the highest-load condition. The average increases in V̇O_2_/kg were 13.2% and 25.2% for $M_{\text{2}}$ and $M_{\text{3}}$, respectively, relative to $M_{\text{1}}$, while the corresponding increases in EEm were 13.2% and 25.4%. These results indicate that metabolic demand in the present experiment was more closely associated with the progressively increasing external resistance than with propulsion velocity alone. In particular, the $M_{\text{3}}$ condition demonstrates that a lower propulsion velocity does not necessarily indicate a lower metabolic demand when the torque required to propel the wheelchair is increased. This observation supports consideration of mechanical power, which incorporates both torque and wheel velocity, rather than velocity alone when describing the external mechanical demand of wheelchair propulsion.

Analysis of Table 3 shows that previous studies [30–33] investigated wheelchair propulsion over generally higher velocity ranges than those observed in the present study. However, direct numerical comparison of metabolic outcomes between studies is limited by substantial methodological differences. In addition to differences in participant characteristics, wheelchair configuration, propulsion protocols, and methods of prescribing exercise intensity, the studies also differed in the definition and calculation of the reported metabolic outcomes. More generally, methods used to determine or estimate energy expenditure differ in their underlying assumptions, applicability, and limitations, which should be considered when comparing metabolic outcomes across studies [34]. Some studies reported physical activity energy expenditure, whereas others reported energy expenditure, metabolic rate, or MET values calculated using study-specific definitions. Consequently, these measures should not be regarded as physiologically interchangeable, and the comparisons presented in Table 3 are intended to be descriptive rather than to demonstrate equivalence between studies. Nevertheless, the reported data consistently illustrate that metabolic demand during wheelchair propulsion depends on more than propulsion velocity alone. The energy expenditure values obtained in the present study overlap with the lower range reported in [3], despite the lower propulsion velocities observed in the present experimental protocol. Differences in fitness level, type and severity of impairment, propulsion technique, wheelchair configuration, and external mechanical resistance may all contribute to differences in metabolic demand between studies. Therefore, wheelchair velocity should not be considered an independent or universally comparable indicator of exercise intensity across different experimental protocols. The present findings can also be compared, with appropriate caution, with those reported in [35], where predictive models of energy expenditure incorporating propulsion power, speed, and heart rate were investigated. The experimental protocol used in [35] involved higher wheelchair velocities and a wider range of heart-rate responses, which limits direct comparison with the present dataset. Nevertheless, both studies support the general concept that wheel-level mechanical power contains relevant information regarding the metabolic demand of wheelchair propulsion. In [35], the association between wheelchair power output and energy expenditure across the investigated intensities was reported as r = 0.69.

In the present study, agreement between measured V̇O_2_/kg values and participant-level LOPO-CV predictions was assessed using repeated-measures Bland–Altman analysis (Fig 3). The analysis yielded a mean bias of −0.156 mL O_2_·kg^−1^·min^−1^ (95% CI: −0.6259 to 0.2022), indicating little overall systematic tendency toward under- or overestimation. However, the limits of agreement ranged from −2.389 mL O_2_·kg^−1^·min^−1^ (95% CI: −3.1667 to −1.7711) to 2.077 mL O_2_·kg^−1^·min^−1^ (95% CI: 1.6096 to 2.6636), demonstrating that uncertainty remained substantial at the level of individual predictions. Therefore, the relatively small mean bias should not be interpreted as evidence that the proposed model is interchangeable with indirect calorimetry. Rather, the Bland–Altman results complement the LOPO-CV performance metrics by showing that, although systematic prediction error was small, individual prediction errors may still be considerable. This finding further supports interpretation of the proposed model as preliminary and highlights the need for external validation in larger independent cohorts.

The final-30-s sensitivity analysis further showed that the main interpretation was not dependent on averaging over the complete 3-min trial. As expected, restricting the analysis to the final 30 s resulted in higher V̇O_2_/kg values, consistent with reduced influence of the initial oxygen-uptake transient. However, the general relationship between mechanical power, heart rate, and mass-specific oxygen uptake remained substantively unchanged, which is important for the overall interpretation of the present study. Cross-validated $R^{\text{2}}$ was virtually identical (0.578 vs 0.575), whereas RMSE, MAE, and the Bland–Altman limits of agreement were larger in the final-30-s analysis. The final 30-s windows also contained relatively few individual breaths, increasing the potential influence of breath-to-breath variability. These findings support retaining the complete-trial analysis as the primary analysis while treating the final-30-s model as a sensitivity analysis rather than as evidence of confirmed physiological steady state.

Previous studies have explored several approaches for estimating physical activity and energy expenditure during wheelchair propulsion, including accelerometer-based systems [36,37], smartphones [38], heart-rate monitoring devices [39], and wheel-mounted systems capable of measuring propulsion torque or power [40]. The present results provide additional support for investigating wheel-level mechanical power as a predictor of metabolic demand. Unlike velocity alone, mechanical power incorporates both the speed of propulsion and the torque required to overcome the imposed mechanical resistance. This distinction appears particularly relevant under conditions such as those observed in $M_{\text{3}}$, where metabolic demand increased despite a reduction in propulsion velocity. Recent studies have also demonstrated the potential of inertial measurement units for estimating mechanical work and power outside conventional laboratory measurement systems [41]. Future studies should investigate whether similar integrated sensing approaches can be adapted to estimate propulsion torque and mechanical power during everyday manual wheelchair use.

At the same time, the present pilot study does not establish that mechanical power is superior to alternative sensor combinations or that the proposed approach is ready for implementation in a practical monitoring device. Rather, the findings indicate that the combination of mechanical power and heart rate warrants further investigation as a basis for estimating metabolic demand during manual wheelchair propulsion. Future studies should evaluate this relationship in larger and more diverse cohorts and under a broader range of propulsion conditions, including real-world environments.

The obtained findings support further investigation of integrated sensor systems capable of determining propulsion torque. Torque measurement, and consequently mechanical power estimation, may provide additional information beyond propulsion velocity alone when estimating metabolic demand under different wheelchair propulsion conditions. This may be particularly relevant when external mechanical demand changes independently of velocity, for example as a result of increased rolling resistance. Future studies should also investigate whether combining mechanical power with upper-limb motion sensor data can further improve the estimation of metabolic demand.

A limitation of the presented study is the relatively small number of participants, which may restrict the generalizability of the findings to the broader population of manual wheelchair users. On the other hand, the study was not limited exclusively to individuals with spinal cord injury (SCI), thereby providing insight into energy expenditure within a more diverse study population. Although the mechanical effects of differences in wheelchair mass, wheel type, tire characteristics, and rolling resistance were reflected in the measured wheel-level mechanical power, the use of participants’ personal wheelchairs may also have influenced propulsion technique, posture, and mechanical efficiency. Therefore, future studies should compare standardized and individually configured wheelchairs to determine whether wheelchair configuration affects the relationship between external mechanical power and metabolic demand. Although the imposed increments in braking resistance were standardized, the resulting absolute and relative mechanical workloads differed between participants. Mechanical power therefore reflects the actual external workload more directly than the nominal braking condition; nevertheless, differences in body mass and functional capacity may have influenced the physiological response to a given absolute power output.

Because propulsion velocity and mechanical power were not standardized across participants, the observed relationships should be interpreted as responses to progressively increasing external resistance under self-selected propulsion conditions rather than as responses to identical absolute workloads. Although this approach preserved individual cadence–stroke mechanics and reduced the risk of artificially modifying propulsion technique, differences in physical fitness, propulsion technique, and mechanical efficiency may have contributed to the variability in metabolic demand.

The three-minute duration of each experimental condition may have been insufficient for complete metabolic steady state to be achieved in all participants. Retrospective analysis showed relatively small median changes in V̇O_2_ and RER during the final minute of the trials; however, greater variation was observed in individual cases. Consequently, the reported metabolic variables should be interpreted as responses averaged over the experimental trial rather than as strictly steady-state values. Future studies should employ longer constant-load stages and prospectively defined V̇O_2_ and RER stability criteria.

Heart rate may be influenced by individual factors such as lesion characteristics, autonomic dysfunction, medication use, and physical fitness. Although lesion level and self-reported physical activity and fitness status were documented, autonomic function and cardiorespiratory fitness were not assessed using standardized clinical or exercise tests. Some participants reported cardiovascular medication use; however, medication effects on heart-rate response were not specifically assessed or controlled. These factors may therefore have contributed to inter-individual variability in the HR–V̇O_2_ relationship.

The experiments were conducted using a wheelchair ergometer under laboratory conditions, which may limit the extent to which the findings can be generalized to real-world wheelchair propulsion. The study was further limited by the small and heterogeneous sample, sex imbalance, fixed propulsion cadence, and repeated-measures design. Although the statistical analyses accounted for repeated observations within participants, the limited number of participant clusters restricts the precision and generalizability of the findings. In addition, individual recovery durations between experimental conditions were not prospectively recorded, and the proposed model has not been externally validated. Therefore, further research should evaluate the model in larger, independent, and more diverse cohorts, including both laboratory and real-world propulsion conditions.

## 5. Conclusions

Within the framework of the present pilot study, a preliminary model was developed to estimate mass-specific oxygen uptake (V̇O_2_/kg) during manual wheelchair propulsion based on mechanical power and heart rate. The findings suggest that these variables may provide useful complementary information for estimating metabolic demand; however, the observed relationships and predictive performance should be interpreted cautiously given the small and heterogeneous study sample. The proposed model has undergone only internal participant-level evaluation using leave-one-participant-out cross-validation and should not be regarded as externally validated.

Further research is required to evaluate the model in larger, independent, and more diverse cohorts and under a broader range of propulsion conditions. Future studies should also investigate methods for practical estimation of user-generated propulsion torque and determine whether the combination of mechanical power and physiological parameters can provide sufficiently robust estimates of metabolic demand for physical activity monitoring during manual wheelchair propulsion.

## Supporting information

S1 Table Detailed demographic, anthropometric, clinical, and wheelchair-related characteristics of the study participants. Completeness of injury was not clinically assessed in all participants.
(DOCX)

S2 TableParticipant-level body mass, mean propulsion torque, mechanical power, and relative mechanical power for conditions $M_{1}-M_{3}$.(DOCX)
