# Supplementary material for: A preliminary model for estimating mass-specific oxygen uptake during manual wheelchair propulsion from mechanical power and heart rate
Source: PLoS One. 2026 Oct 6;21(10):e0359323. doi: 10.1371/journal.pone.0359323 (PMC13641715; doi:10.1371/journal.pone.0359323)
Supplement: Supplementary file 1 [file pone.0359323.s001.docx]

**S1 Table. Detailed demographic, anthropometric, clinical, and wheelchair-related characteristics of the study participants.** Completeness of injury was not clinically assessed in all participants.

| Participant | Sex | Height (m) | Weight (kg) | Wheelchair weight (kg) | Body mass index (kg/m² ) | Upper limb length (m) | Diagnosis | Etiology | Duration of disability | Years of wheelchair use | Functional classification | Degree of disability | Physical activity level | Medication use |
| --- | --- | --- | --- | --- | --- | --- | --- | --- | --- | --- | --- | --- | --- | --- |
| A1 | M | 1.8 | 80 | 7 | 24.7 | 0.56 | Locomotor impairment or musculoskeletal impairment (Code 05-R); Neurological disorders (Code 10-N); Paraplegia | Non-injury | Since birth | 30 | Independent manual wheelchair user; assistance from another person required | Severe | Low | Telmisartan |
| A2 | F | 1.58 | 47 | 7.5 | 18.8 | 0.49 | Locomotor impairment or musculoskeletal impairment (Code 05-R); Neurological disorders (Code 10-N); Paraplegia | Non-injury | Since birth | 26 | Independent manual wheelchair user; assistance from another person required | Severe | Low | N/A |
| A3 | M | 1.68 | 80 | 12 | 28.3 | 0.48 | Locomotor impairment or musculoskeletal impairment (Code 05-R); Neurological disorders (Code 10-N); Paraplegia | Injury | 19 | 19 | Independent manual wheelchair user; assistance not required | Severe | High | Furaginum |
| A4 | M | 1.77 | 90 | 12 | 28.7 | 0.59 | Locomotor impairment or musculoskeletal impairment (Code 05-R); Neurological disorders (Code 10-N); Paraplegia | Injury | 20 | 20 | Independent manual wheelchair user; assistance from another person required | Severe | Low | Inhaled corticosteroids, ACE inhibitors |
| A5 | F | 1.65 | 70 | 8 | 25.7 | 0.52 | Locomotor impairment or musculoskeletal impairment (Code 05-R); Tetraplegia with predominant lower limb involvement | Non-injury | Since birth | 20 | Independent manual wheelchair user; assistance from another person required | Severe | Low | N/A |
| A6 | M | 1.52 | 78 | 5 | 33.8 | 0.46 | Neurological disorders (Code 10-N) | Non-injury | Since birth | 28 | Independent manual wheelchair user; assistance from another person required | Severe | Low | N/A |
| A7 | F | 1.81 | 84 | 8 | 25.6 | 0.60 | Neurological disorders (Code 10-N) | Non-injury | Since birth | 30 | Independent manual wheelchair user; assistance not required | Moderate | Medium | N/A |
| A8 | M | 1.72 | 94 | 6 | 31.8 | 0.60 | Neurological disorders (Code 10-N), Genitourinary disorders (Code 09-M); Spina bifida with myelomeningocele | Non-injury | Since birth | 26 | Independent manual wheelchair user; assistance from another person required | Severe | Low | Dexilant |
| A9 | M | 1.87 | 84 | 12 | 24.0 | 0.67 | Locomotor impairment or musculoskeletal impairment (Code 05-R); Neurological disorders (Code 10-N); Paraplegia | Non-injury | Since birth | 27 | Independent manual wheelchair user; assistance not required | Moderate | High | N/A |
| A10 | M | 1.73 | 130 | 12 | 43.4 | 0.55 | Locomotor impairment or musculoskeletal impairment (Code 05-R); Paraplegia | Non-injury | Since birth | 31 | Independent manual wheelchair user; assistance not required | Moderate | High | N/A |
