# Supplementary material for: A preliminary model for estimating mass-specific oxygen uptake during manual wheelchair propulsion from mechanical power and heart rate
Source: PLoS One. 2026 Oct 6;21(10):e0359323. doi: 10.1371/journal.pone.0359323 (PMC13641715; doi:10.1371/journal.pone.0359323)
Supplement: Supplementary file 2 [file pone.0359323.s002.docx]

**S2 Table. Participant-level body mass, mean propulsion torque, mechanical power, and relative mechanical power for conditions** $\boldsymbol{M}_{\mathbf{1}}\boldsymbol{-}\boldsymbol{M}_{\mathbf{3}}$

| Participant | Body mass  (kg) | $\boldsymbol{M}_{\mathbf{1}}$  torque (Nm) | $\boldsymbol{M}_{\mathbf{1}}$  power  (W) | $\boldsymbol{M}_{\mathbf{1}}$ relative power (W/kg) | $\boldsymbol{M}_{\mathbf{2}}$ torque (Nm) | $\boldsymbol{M}_{\mathbf{2}}$ power (W) | $\boldsymbol{M}_{\mathbf{2}}$ relative power (W/kg) | $\boldsymbol{M}_{\mathbf{3}}$ torque (Nm) | $\boldsymbol{M}_{\mathbf{3}}$ power (W) | $\boldsymbol{M}_{\mathbf{3}}$ relative power (W/kg) |
| --- | --- | --- | --- | --- | --- | --- | --- | --- | --- | --- |
| A1 | 80 | 1.618 | 1.074 | 0.013 | 1.383 | 1.052 | 0.013 | 3.040 | 2.023 | 0.025 |
| A2 | 47 | 1.363 | 1.128 | 0.024 | 1.522 | 1.164 | 0.025 | 1.663 | 1.141 | 0.024 |
| A3 | 80 | 1.399 | 1.749 | 0.022 | 1.591 | 1.955 | 0.024 | 1.797 | 1.948 | 0.024 |
| A4 | 90 | 1.464 | 2.016 | 0.022 | 2.283 | 2.994 | 0.033 | 2.339 | 2.663 | 0.030 |
| A5 | 70 | 1.549 | 2.249 | 0.032 | 2.290 | 3.099 | 0.044 | 2.425 | 2.815 | 0.040 |
| A6 | 78 | 2.034 | 3.037 | 0.039 | 2.306 | 3.630 | 0.047 | 2.984 | 3.746 | 0.048 |
| A7 | 84 | 2.261 | 4.109 | 0.049 | 2.382 | 4.241 | 0.050 | 3.800 | 5.717 | 0.068 |
| A8 | 94 | 2.317 | 4.470 | 0.048 | 2.589 | 4.641 | 0.049 | 4.005 | 6.230 | 0.066 |
| A9 | 84 | 2.321 | 5.092 | 0.061 | 2.694 | 6.963 | 0.083 | 5.705 | 9.711 | 0.116 |
| A10 | 130 | 2.490 | 7.728 | 0.059 | 3.577 | 10.906 | 0.084 | 5.845 | 18.293 | 0.141 |
